# Nomogram-based prognostic tool for stage IIIB/IV non-small cell lung cancer patients undergoing traditional Chinese medicine treatment

**DOI:** 10.1016/j.heliyon.2024.e31449

**Published:** 2024-05-18

**Authors:** Yihong Liu, Haochuan Ma, Rui Zhou, Yadong Chen, Yanjuan Zhu, Xuesong Chang, Jicai Chen, Haibo Zhang

**Affiliations:** a Department of Oncology, The Second Affiliated Hospital of Guangzhou University of Traditional Chinese Medicine, Guangdong Provincial Hospital of Chinese Medicine, Guangzhou, Guangdong, China; b Guangdong Provincial Hospital of Chinese Medicine Postdoctoral Research Workstation, Guangzhou, Guangdong, China; c Department of Thoracic Surgery, The Second Affiliated Hospital of Guangzhou University of Traditional Chinese Medicine, Guangdong Provincial Hospital of Chinese Medicine, Guangzhou, China

**Keywords:** Nomogram, Overall survival, Non-small cell lung cancer, Traditional Chinese medicine, Prognosis

## Abstract

**Objective:**

Given the significant impact of long-term traditional Chinese medicine (TCM) treatment on the prognosis of patients with non-small cell lung cancer (NSCLC), we aimed to develop nomograms, with or without consideration of TCM treatment duration, to accurately predict the overall survival (OS) of patients with stage IIIB/IV NSCLC treated with TCM.

**Methods:**

Nomograms were developed from a training cohort comprised of 292 patients diagnosed with NSCLC, using univariate and multivariate Cox regression analyses to screen for various prognostic factors with and without TCM treatment. The nomograms were evaluated using the concordance index (*C*-index), calibration curve, and decision curve analysis (DCA), after which they were validated, using the bootstrap self-sampling method for internal validation, and a validation cohort comprised of 175 patients for external validation. Bootstrap validation is a resampling technique that involves randomly selecting and replacing data from the original dataset to make statistical inferences, thereby circumventing the issue of sample reduction that can arise from cross-validation.

**Results:**

We identified seven significant prognostic factors for OS. For nomogram A (excluding TCM treatment time), the *C*-indexes (95 % confidence interval [CI]) were 0.674 (0.635–0.712) and 0.660 (0.596–0.724) for the training and validation cohorts, respectively. For nomogram B (including TCM treatment time), the *C*-indices (95 % CI) were 0.846 (0.822–0.870) and 0.783 (0.730–0.894), for the training and validation cohorts, respectively, indicating that nomogram B was superior to nomogram A. Both the calibration curves and DCA results exhibited favorable clinical concordance and usefulness.

**Conclusion:**

The nomogram B yielded precise prognostic predictions for patients with advanced NSCLC treated with TCM.

## 1 Introduction

In recent years, there has been an upward trend in the prevalence of cancer worldwide, with lung cancer ranking as the leading cause of cancer deaths globally, accounting for approximately 2.2 million new cases and 1.8 million deaths in 2020 [1,2]. Non-small cell lung cancer (NSCLC) is the predominant pathological type of lung cancer, for which current therapeutic strategies include surgical resection, radiotherapy, chemotherapy, targeted therapy, and immunotherapy [3,4]. Patients, however, are often unable to undergo surgery as a result of the advanced stages of the tumor or presence of metastases. Therefore, it is imperative to improve the survival rate and quality of life (QOL) of patients with advanced NSCLC [5,6].

Traditional Chinese medicine (TCM), which is extensively utilized and embraced in China, consists of a variety of treatments, including acupuncture, Chinese herbal remedies, and manipulative treatments. It serves as an essential component in the prevention and treatment of NSCLC in China, mitigating the adverse effects of radio and chemotherapy, and reducing the risk of recurrence [7,8]. Clinical studies have provided evidence that the use of TCM in the treatment of advanced NSCLC improves both patient survival and QOL [[9], [10], [11]]. Wang et al. [10] found that patients with stage II–III NSCLC who underwent TCM treatment for at least three months had improved two-year survival rates. Another study found that long-term TCM treatment was an independent protective factor affecting the prognosis of patients with NSCLC [12], suggesting that TCM treatment may be an independent prognostic factor for survival in cases of advanced NSCLC. Few studies, however, have used multivariate Cox regression analyses to determine survival prognostic factors in patients with advanced NSCLC who were treated with TCM. Luo et al. created a nomogram to predict progression-free survival (PFS) in patients undergoing adjuvant TCM treatment for NSCLC. Their study, however, did not focus on patients with advanced-stage disease nor on overall survival (OS) [13]. The present study, therefore, aimed to evaluate the prognostic factors affecting OS in patients with NSCLC treated with TCM, and to establish a nomogram for accurate prognostication, providing evidence for the use of TCM in NSCLC treatment.

In the present retrospective study of patients with advanced NSCLC undergoing treatment with TCM, we initially extracted prognostic factors through a comprehensive search of nine Chinese and English electronic databases, as well as through manual examination. Additionally, we developed two predictive nomograms – nomogram A (defined as excluding the time of TCM treatment) and nomogram B (defined as including the time of TCM treatment) – based on these prognostic factors to assess OS, and subsequently performed independent internal and external validations.

## 2 Methods

### 2.1 Patients enrolled

**Fig. 1:**
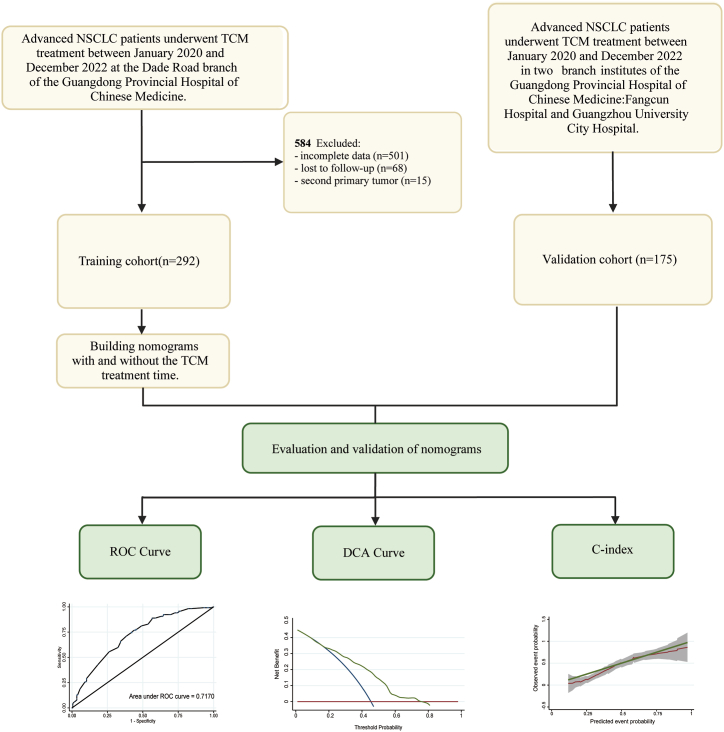
Flow chart of study procedures. Abbreviations: NSCLC, Non-small cell lung cancer; TCM, Traditional Chinese Medicine.

The present study involved a retrospective analysis of 467 patients diagnosed with advanced (stage IIIB or IV) NSCLC at three branches of Guangdong Provincial Hospital of Chinese Medicine (GPHCM) in Guangzhou, China. Patients were recruited from Dade Road, Fangcun, and Guangzhou University City Hospitals from January 2020 to December 2022. A flowchart of the study design is shown in Fig. 1. The ethics Committee of GPHCM reviewed and approved the protocol for the present study. The need for informed consent was waived, due to the retrospective nature of the present study.

The inclusion criteria for the present study were as follows: 1) patients diagnosed with NSCLC through tissue biopsy from primary or metastatic lesions, or by obtaining cellular specimens from sputum, plasma luminal fluid, or other sources, histological subtyping of the lung tumors was conducted based on 2021 World Health Organization (WHO) criteria; 2) patients had a clinical stage of NSCLC, grade IIIB–IV, based on the American Joint Committee of Cancer (AJCC) 8th edition Cancer Staging Manual; 3) age >18 years; and 4) patients had normal kidney and liver function.

The exclusion criteria for the present study were as follows: 1) patients received any anti-tumor treatment prior to enrollment; 2) patients were lactating or pregnant; 3) patients had a second primary tumor or the primary tumor was not NSCLC; and 4) patients with incomplete follow-up or clinical data.

### 2.2 Follow-up

All patients were underwent follow up via telephone and outpatient visits, from one day after receiving a histologically or cytologically confirmed diagnosis until death or the last follow-up (August 2023). The following information was collected during follow-ups: general condition of the patient; cancer treatment; disease progression; survival status (alive/dead); reason for death; and survival time. The follow-up endpoint was OS, which was defined as the time from diagnosis to death or the final follow-up [14].

### 2.3 Selection criteria for predictor variables

To comprehensively determine all relevant prognostic factors for NSCLC, we systematically searched the following Chinese and English electronic databases through August 2023, the China National Knowledge Infrastructure (CNKI); Chinese BioMedical Literature (CBM); Chinese Science Journal Full Text (CQVIP); Wanfang database; PubMed; Embase; Cochrane Library; Scopus; and Web of Science. The following were determined to be potential prognostic factors after the initial search, as seen in Table S1: patient demographic information (sex, age, smoking index, smoking status, body mass index, blood type, physical status, and family history), oncologic characteristics (including tumor-node-metastasis stage, pathological type(s), differentiation status, gene mutation, metastatic site, and comorbidities); treatment-related indicators (overall treatment regimen, maintenance therapy, treatment line, TCM treatment regimen, and the length of the TCM treatment); blood biochemistry indicators (tumor markers, routine blood count, coagulation indicators, and kidney and liver function tests); and survival indices.

### 2.4 TCM therapy

In the present study, TCM therapy predominantly consisted of acupuncture and Chinese herbal remedies, which included a range of commonly used Chinese medicines, such as Baizhu (*Atractylodes macrocephala Koidz*), Fuling (*Poria cocos*), Huangqi (*Astragali Radix*), and Banxia (*Pinellia ternate*). Various formulas, such as Shashen-Maidong and Er-Chen decoctions are utilized in TCM, as are Chinese patent medicines, including *Taxus chinensis* capsules, *Kanglaite* injections, *Brucea javanica* oil emulsion, *Cinobufacin* capsules, and *Xiao'aiping* tablets.

### 2.5 Statistical analyses

To construct and validate the nomograms in the present study, patients were randomly divided into training and validation cohorts, as follows: 292 patients from Dade Road Hospital comprised the training cohort, and 175 patients from Fangcun and Guangzhou University City Hospitals comprised the independent external validation cohort. In the training cohort, univariate Cox regression analyses were conducted using the Kaplan-Meier “survival” package of R version 4.2.3 to initially screen and select predictive features that were useful for predicting OS [15]. Furthermore, several supplementary R packages, namely “rms,” “VIM,” “pec,” “randomForestSRC,” and “foreign,” were also utilized in the present study. Variables that yielded statistically significant results (*P* < 0.05) in the univariate analyses were subsequently included in the multivariate Cox regression analysis. A nomogram was developed using the prognostic factors identified in these analyses to predict the 1 and 3-year survival probabilities of patients diagnosed with advanced NSCLC.

The accuracy of the nomograms was then evaluated using various methods, including the concordance index (*C*-index) via the bootstrap self-sampling method, time-dependent receiver operating characteristics (ROC), decision curve analysis (DCA), and calibration curves. The *C*-index values, ranging from 0.5 to 1.0, were used to categorize the predictive accuracy into low (0.5–0.7), moderate (0.7–0.9), and high (>0.9) levels. The calibration curve method assessed predictive accuracy by comparing the projected survival time derived from the nomograms with the actual survival time of the patient. The DCA was used to predict the clinical usefulness and benefits of the nomograms, based on their clinical accuracy, practicability, and effectiveness, and the bootstrap method was used for resampling, with the number of repetitions set to 1000 for internal validation [[16], [17], [18]].

Statistical analysis, development, evaluation, and verification of the models were performed using SPSS (version 26.0 for Windows), Stata (Stata SE, version 15), and R software version 4.2.3. X-tile (version 3.6.1) was used to analyze the suitable cut off points for the time of TCM treatment [19]. Patient-related data were double-entered and checked using Epidata 3.1. Statistical significance was set at *P* < 0.05 (two-tailed).

## 3 Results

### 3.1 Patients’ baseline characteristics

Based on the aforementioned inclusion criteria, we selected 467 patients for the present study, 292 of which were in the training set (Dade Road Hospital) and while the remaining 175 were in the validation set (Fangcun and Guangzhou University City Hospitals). The majority of patients in the training set were male (193; 66.1 %), former or current smokers (163; 55.8 %), and had adenocarcinomas (237; 81.1 %). A significant proportion of patients received TMC treatment, which mostly involved oral administration (n = 288; 98.6 %). The details are listed in Table 1.

**Table 1:** Demographic and clinical characteristics in the training cohort.

Characteristic	Mean ± SD/n (%)	Characteristic	n (%)	Characteristic	n (%)
**Age (years)**	62.64 ± 11.36	**Sex** Male Female	193 (66.10 %) 99 (33.90 %)	**Family history** Yes No	46 (15.75 %) 246 (84.25 %)
**BMI** <18.5 18.5–23.9 ≥24	45 (15.41 %) 185 (63.36 %) 62 (21.23 %)	**Smoke** Yes No	163 (55.82 %) 129 (44.18 %)	**Smoking index** ≤200 200-400 ≥400	142 (48.63 %) 9 (3.08 %) 141 (48.29 %)
**Blood types** A B O AB Unknow	46 (15.75 %) 59 (20.21 %) 75 (25.68 %) 14 (4.79 %) 98 (33.56 %)	**ECOG PS score** 0 1 2 3 4	18 (6.16 %) 192 (65.75 %) 58 (19.86 %) 22 (7.53 %) 2 (0.68 %)	**Pathological type** Adenocarcinomas Squamous carcinomas Adenosquamous carcinomas Large cell carcinoma Carcinoma	237 (81.16 %) 41 (14.04 %) 6 (2.05 %) 3 (1.03 %) 5 (1.71 %)
**Pathological differentiation** Unknow Highly differentiated Moderately differentiated Poorly differentiated Medium and low differentiation	196 (67.12 %) 4 (1.37 %) 18 (6.16 %) 71 (24.32 %) 3 (1.03 %)	**EGFR mutation** Unknown Wild-type Mutated	105 (35.96 %) 120 (41.1 %) 67 (22.95 %)	**TNM Stage** IIIB IIIC IV	36 (12.33 %) 2 (0.68 %) 254 (86.99 %)
**Stage T** T1 T2 T3 T4	25 (8.56 %) 86 (29.45 %) 43 (14.73 %) 138 (47.26 %)	**Stage N** N0 N1 N2 N3	28 (9.59 %) 17 (5.82 %) 111 (38.01 %) 136 (46.58 %)	**Stage M** M0 M1	38 (13.01 %) 254 (86.99 %)
**Intrapulmonary metastatic** Yes No	124 (42.47 %) 168 (57.53 %)	**Liver metastases** Yes No	39 (13.36 %) 253 (86.64 %)	**Bone metastases** Yes No	131 (44.86 %) 161 (55.14 %)
**Brain metastases** Yes No	70 (23.97 %) 222 (76.03 %)	**Adrenal metastasis** Yes No	31 (10.62 %) 261 (89.38 %)	**Pleural metastasis** Yes No	55 (18.84 %) 237 (81.16 %)
**Comorbidities** Yes No	155 (53.08 %) 137 (46.92 %)	**Pleural effusion** Yes No	105 (35.96 %) 187 (64.04 %)	**Targeted therapy** Yes No	123 (42.12 %) 169 (57.88 %)
**Chemotherapy** Yes No	165 (56.51 %) 127 (43.49 %)	**Radiotherapy** Yes No	25 (8.56 %) 267 (91.44 %)	**Maintenance therapy** Yes No	37 (12.67 %) 255 (87.33 %)
**The number of treatment lines** 0 1 2 3 4 5 6	51 (17.47 %) 146 (50.00 %) 49 (16.78 %) 28 (9.59 %) 10 (3.42 %) 1 (0.34 %) 7 (2.40 %)	**Types of Western medicine treatment** 1 2 3	225 (77.05 %) 58 (19.86 %) 9 (3.08 %)	**TCM treatment** Yes No	288 (98.63 %) 4 (1.37 %%)
**Oral administration of TCM** Yes No	275 (94.18 %) 17 (5.82 %)	**External use of TCM** Yes No	106 (36.30 %) 186 (63.70 %)	**TCM IV infusion** Yes No	184 (63.01 %) 108 (36.99 %)
**Acupuncture** Yes No	112 (38.36 %) 180 (61.64 %)				

Abbreviations: BMI, Body Mass Index; ECOG; Eastern Cooperative oncology Group; EGFR, Epidermal growth factor receptor; IV, Intravenous; TCM, Traditional Chinese Medicine.

### 3.2 Classification of TCM treatment time

After analyzing the data in X-tile, it was found that the ideal cutoff points for TCM treatment time were 164 and 296.18 days (Fig. S1). Consequently, the duration of TCM treatment was subsequently categorized as follows: (1) < 164 days; (2) 164–296.18 days; and (3) > 296.18 days. According to the data presented in Table 1, of the 292 patients in the training set, only 50 received TCM treatment, resulting in a median OS of 3.8 months. Of note, however, the duration of TCM treatments varied widely, which could cause discrepancies in clinical practice and make it challenging to interpret results when categorized based on a cutoff point. Therefore, to elucidate the relationship between the duration of the time of TCM treatment time and OS, we incorporated this variable as a continuous entity in the model and adjusted the unit of measurement from daily to monthly increments.

### 3.3 Screening for prognostic factors

The results of the univariate and multivariate analyses are presented in Table 2. According to the univariate Cox regression analysis, 22 variables were potential prognostic factors for stage IIIB/IV NSCLC, which were then included in the multivariate Cox regression analysis. Additionally, the following seven independent prognostic factors were associated with the prediction of OS in the multivariate analysis: sex; epidermal growth factor receptor (EGFR) gene mutations; adrenal metastases; time of TCM treatment; TCM intravenous (IV) infusion; white blood cell (WBC) count; and maintenance therapy. Among these seven independent prognostic factors, the differentiation of prognoses based on sex and EGFR mutations underscores the influence of individual differences on OS. The inclusion of adrenal metastases highlights the prognostic significance of metastatic patterns, whereas the specific aspects of TCM treatment and maintenance therapy, both in terms of duration and delivery method, underline the effects of cancer treatment on OS. Additionally, the evaluation of the WBC count assesses the impact of the patient's immune status.

**Table 2:** Univariate and multivariate analyses for screening the risk factors in the training cohort.

	Univariate		Multivariate	
	HR (95%CI)	*P*	HR (95%CI)	*P*
Age	1.015 (1.002–1.028)	**0.020**	0.998 (0.983–1.014)	0.811
Sex	0.491 (0.365–0.66)	< **0.001**	0.504 (0.341–0.809)	**0.005**
Family history	0.992 (0.682–1.44)	0.965		
BMI	0.638 (0.509–0.8)	< **0.001**	0.995 (0.772–1.281)	0.966
Smoke	1.653 (1.254–2.18)	< **0.001**	0.889 (0.440–1.796)	0.742
Smoking index	1.374 (1.194–1.58)	< **0.001**	0.906 (0.643–1.277)	0.574
Blood types				
A	Reference			
B	0.998 (0.65–1.53)	0.993		
O	1.111 (0.753–1.64)	0.595		
AB	1.228 (0.865–1.75)	0.251		
Unknow	1.202 (0.651–2.22)	0.556		
ECOG PS score	1.473 (1.23–1.76)	< **0.001**	1.130 (0.907–1.410)	0.276
**Pathological type**				
Adenocarcinomas	Reference			
Squamous carcinomas	0.639 (0.262–1.56)	0.325		
Adenosquamous carcinomas	0.859 (0.333–2.22)	0.754		
Large cell carcinoma	0.6 (0.161–2.24)	0.447		
Carcinoma	1.701 (0.404–7.15)	0.469		
**Pathological differentiation**	1.111 (1.005–1.23)	**0.039**	1.008 (0.901–1.129)	0.885
**EGFR mutation**				
Unknow	Reference			
Wild-type	2.419 (1.654–3.54)	< **0.001**	1.394 (0.866–2.246)	0.172
Mutated	2.155 (1.476–3.15)	< **0.001**	1.917 (1.168–3.144)	**0.010**
**TNM Stage**	1.018 (0.833–1.24)	0.860		
T	1.098 (0.965–1.25)	0.157		
N	1.037 (0.894–1.2)	0.634		
M	1.043 (0.696–1.56)	0.838		
**Intrapulmonary metastatic**	1.062 (0.807–1.4)	0.666		
**Liver metastases**	1.509 (1.005–2.27)	**0.047**	1.475 (0.936–2.323)	0.094
**Bone metastases**	1.26 (0.961–1.65)	0.095		
**Brain metastases**	1.109 (0.813–1.51)	0.513		
**Adrenal metastasis**	2.535 (1.667–3.85)	< **0.001**	1.688 (1.050–2.715)	**0.031**
**Pleural metastasis**	0.931 (0.655–1.32)	0.689		
**Comorbidities**	1.119 (0.853–1.47)	0.418		
**Pleural effusion**	1.13 (0.854–1.5)	0.393		
**Targeted therapy**	0.57 (0.431–0.75)	< **0.001**	0.809 (0.548–1.195)	0.287
**Chemotherapy**	1.035 (0.784–1.37)	0.806		
**Radiotherapy**	0.691 (0.439–1.09)	0.111		
**Maintenance therapy**	0.521 (0.34–0.8)	**0.003**	0.695 (0.424–1.141)	**0.023**
**Types of Western medicine treatment**	0.667 (0.513–0.87)	**0.003**	1.148 (0.783–1.684)	0.478
**The number of treatment lines**	0.814 (0.731–0.91)	< **0.001**	0.982 (0.825–1.169)	0.837
**TCM treatment**	0.871 (0.278–2.72)	0.812		
**TCM treatment time**	0.997 (0.996–1)	< **0.001**	0.996 (0.996–0.997)	< **0.001**
**Oral administration of TCM**	1.296 (0.663–2.53)	0.448		
**External use of TCM**	1 (0.758–1.32)	0.998		
**TCM IV infusion**	1.415 (1.053–1.9)	**0.021**	1.834 (1.300–2.587)	**0.001**
**Acupuncture**	0.9 (0.683–1.19)	0.453		
**CEA**	1.053 (0.959–1.16)	0.280		
**WBC**	1.532 (1.158–2.03)	**0.003**	1.256 (0.901–1.753)	**0.001**
**RBC**	0.873 (0.683–1.12)	0.281		
**HGB**	0.996 (0.988–1)	0.347		
**PLT**	1.001 (0.999–1)	0.322		
**ALT**	0.999 (0.994–1)	0.696		
**ATS**	1.449 (0.907–2.32)	0.121		
**TBil**	1.019 (0.98–1.06)	0.343		
**DBil**	2.653 (1.443–4.88)	**0.002**	1.212 (0.599–2.451)	0.593
**TP**	0.991 (0.976–1.01)	0.250		
**Alb**	0.567 (0.423–0.76)	< **0.001**	0.843 (0.602–1.181)	0.322
**GGT**	1.337 (1.041–1.72)	**0.023**	1.099 (0.810–1.491)	0.545
**ALP**	1 (0.999–1)	0.448		
**PT**	1.975 (1.32–2.96)	**0.001**	1.270 (0.690–2.338)	0.442
**FIB**	1.276 (0.925–1.76)	0.138		
**INR**	2.206 (1.253–3.88)	**0.006**	1.025 (0.437–2.403)	0.954
**APTT**	1.013 (0.985–1.04)	0.382		

Abbreviations: BMI, Body Mass Index; ECOG; Eastern Cooperative oncology Group; EGFR, Epidermal growth factor receptor; IV, Intravenous; TCM, Traditional Chinese Medicine; HR, Hazard ratio; CEA, Carcinoembryonic antigen; WBC, White blood cell; RBC, Red Blood Cell; HGB, Hemoglobin; PLT; Platelet; ALT, Alanine aminotransferase; AST, Aspartate aminotransferase; TBil, Total bilirubin; DBil, Direct bilirubin; TP, Total protein; Alb, Albumin; GGT, γ-glutamyl transpeptidase; ALP, Alkaline phosphatase; PT, Prothrombin time; FIB, Fibrinogen; INR, International normalized ratio; APTT, Activated partial thromboplastin time.

### 3.4 Nomogram construction

**Fig. 2:**
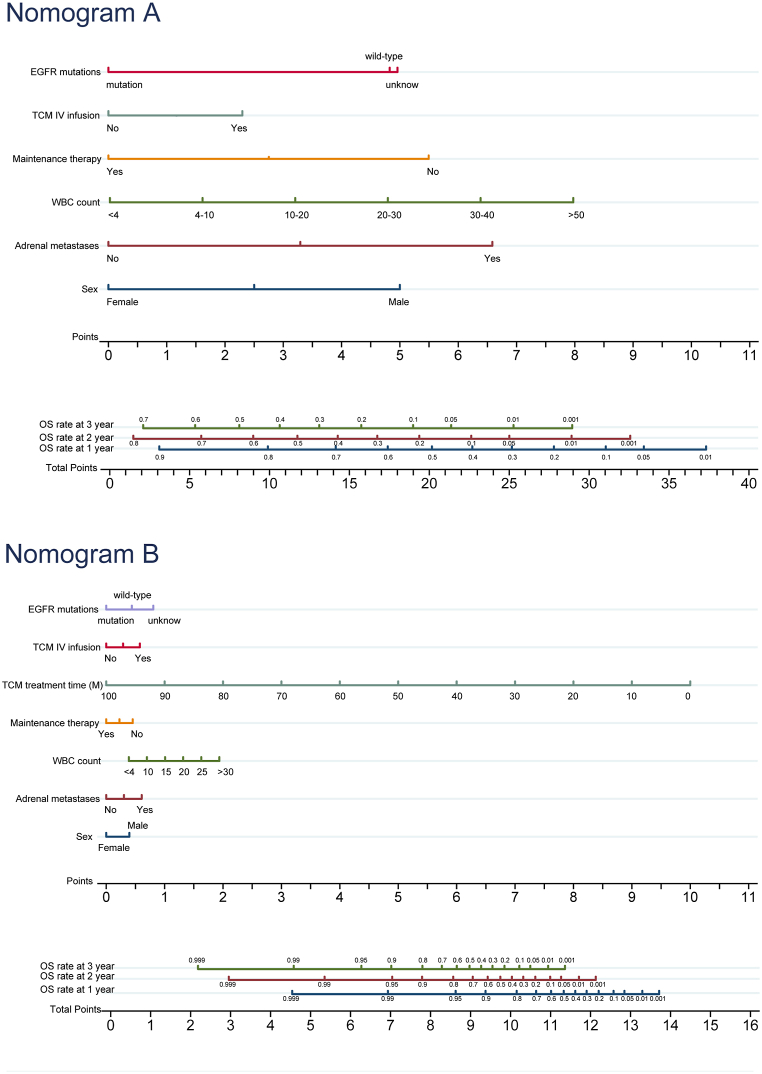
Nomograms for predicting 1-, 2-, and 3-year OS rate in advanced NSLCL patients: (**A**) excluding the TCM treatment time; (**B**) including the TCM treatment time. Abbreviations: OS, Overall survival; NSCLC, Non-small cell lung cancer; TCM, Traditional Chinese Medicine; EGFR, Epidermal growth factor receptor; IV, Intravenous; WBC, White blood cell; M, Month.

Two nomograms were constructed using the aforementioned prognostic factors: nomogram A, which excluded the time of TCM treatment, and nomogram B, which incorporated the time of TCM treatment (Fig. 2). Nomogram A revealed that WBC count had the most significant influence on OS, followed by adrenal metastases, sex, maintenance therapy, EGFR gene mutations, and TCM IV infusion. Nomogram B, however, revealed that TCM treatment time significantly influenced OS, followed by WBC count, adrenal metastases, sex, TCM IV infusion, maintenance therapy, and EGFR gene mutations. Each prognostic variable was assigned a score on a scale, and the cumulative scores were subsequently obtained by totaling the scores of the selected variables. Based on the patient data, nomograms can assist in estimating the OS of individual patients.

### 3.5 Nomogram evaluation

**Fig. 3:**
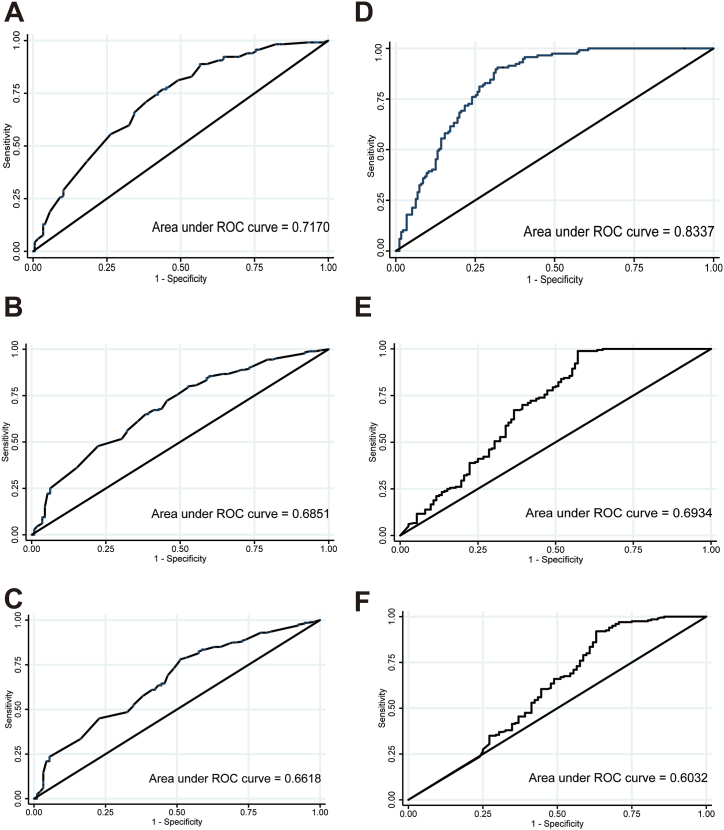
ROC curves and AUC for Nomogram A and Nomogram B. **A-C**: ROC curves for 1-year (A), 2-year (B), and 3-year (C) OS rate for Nomogram A. ***E*-F**: ROC curves for 1-year (D), 2-year (E), and 3-year (F) OS rate for Nomogram B. Abbreviations: ROC, Receiver operating characteristics; AUC, Area under curve; OS, Overall survival.

**Fig. 4:**
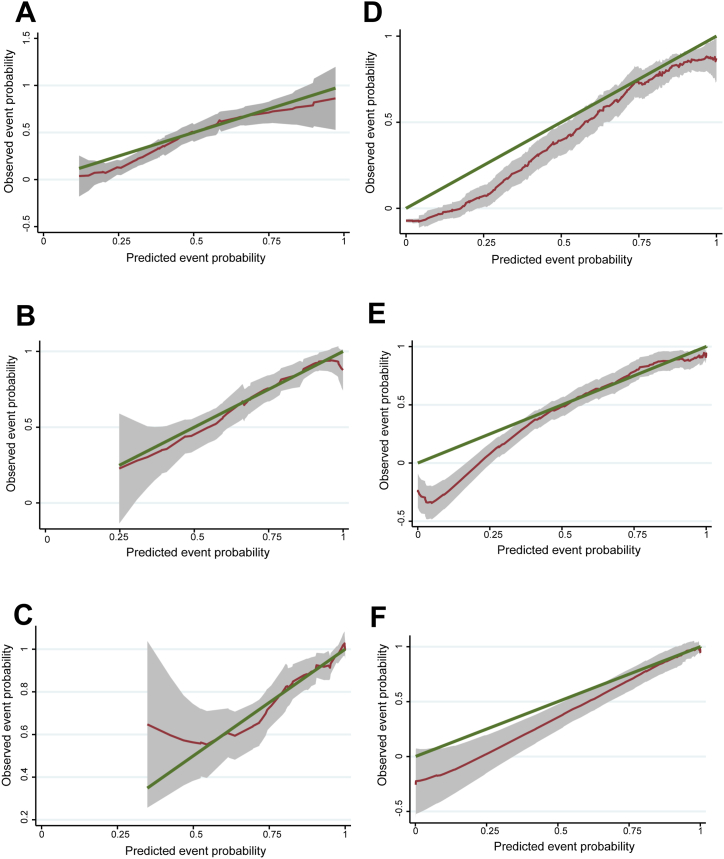
Calibration curves for Nomogram A and Nomogram B. **A-C**: Calibration curves for 1-year (A), 2-year (B), and 3-year (C) OS rate for Nomogram A. ***E*-F**: Calibration curves for 1-year (D), 2-year (E), and 3-year (F) OS rate for Nomogram B. Abbreviations: OS, Overall survival.

**Fig. 5:**
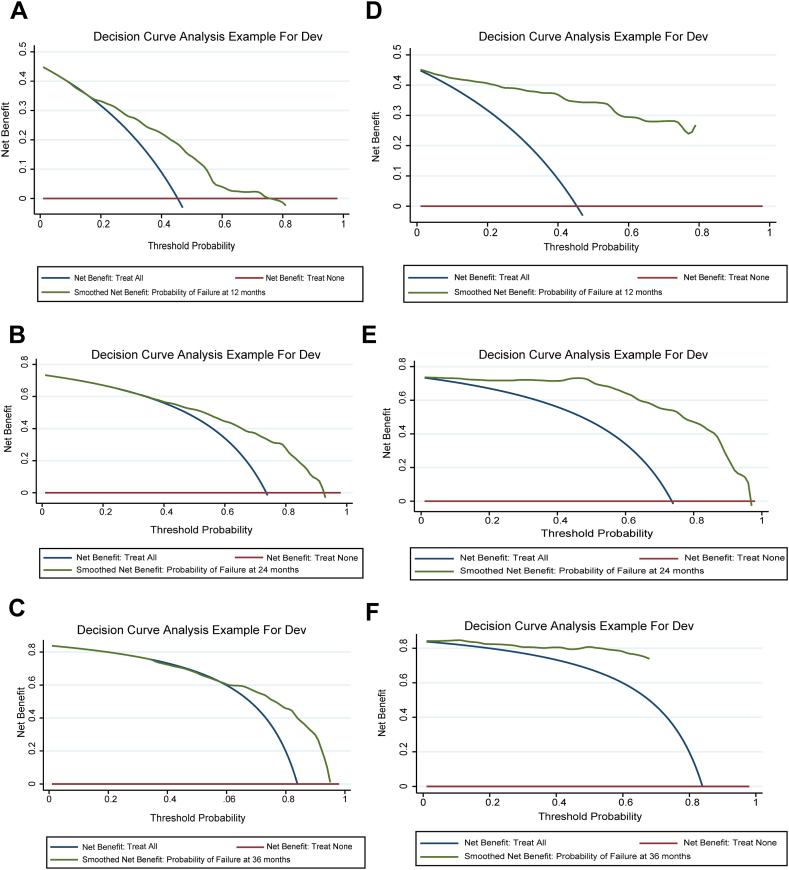
DCA for Nomogram A and Nomogram B. **A-C**: DCA for 1-year (A), 2-year (B), and 3-year (C) OS rate for Nomogram A. ***E*-F**: DCA for 1-year (D), 2-year (E), and 3-year (F) OS rate for Nomogram B. Abbreviations: DCA, Decision curve analysis; OS, Overall survival.

The *C*-index served as a key metric for quantifying the predictive accuracy of each nomogram. For nomogram A, the *C*-index was 0.674 (95 % confidence interval [CI], 0.635–0.712), suggesting a moderate predictive power. Nomogram B, however, exhibited a significantly higher *C*-index, at 0.846 (95 % CI, 0.822–0.870), indicating superior accuracy in predicting patient outcomes. The areas under the ROC curve (AUCs) for the 1-, 2-, and 3-year OS rates were 0.717, 0.685, and 0.662, respectively. For nomogram B, the corresponding AUCs were 0.834, 0.693, and 0.603 (Fig. 3). Calibration plots, as seen in Fig. 4, offer a visual comparison between predicted outcomes and actual survival rates, revealing a closer alignment with nomogram A. DCA, illustrated in Fig. 5, assesses the clinical applicability of each nomogram. The analysis of nomogram B indicated a higher net benefit across a wide range of threshold probabilities, confirming its broader clinical validity and utility despite calibration differences.

### 3.6 Internal validation of nomogram

The training cohort underwent internal validation using the computer resampling bootstrap technique, which involved reconstructing nomograms A and B for 1000 iterations. The *C*-indices were 0.654 and 0.845 for nomograms A and B respectively, representing a significantly better differentiation of nomogram B than A. The calibration plots indicated that following internal reconstruction, the survival outcomes depicted in nomogram An exhibit a greater level of adherence to the observed survival rates than nomogram B (Fig. S2).

### 3.7 External validation of nomograms

External validation was conducted using data from the validation cohort, revealing a *C*-index of 0.660 (95 % CI, 0.596–0.724) for nomogram A and 0.783 (95 % CI, 0.730–0.894) for nomogram B. Fig. S3 shows that there was a higher level of concordance between the predicted and actual values in nomogram A than B.

## 4 Discussion

The present study utilized nomograms to predict the OS of patients with stage IIIB/IV NSCLC who underwent TCM treatment. A total of 467 patients were included in the present study. Through univariate and multivariate analyses of the training cohort, seven prognostic factors were subsequently used to construct nomograms, which were then analyzed via internal and external validation, with and without TCM treatment time. To the best of the authors’ knowledge, the present is the first study to build a survival prediction model for patients with stage IIIB/IV NSCLC that incorporates TCM treatment time. The resulting model effectively forecasts the survival outcomes of patients with NSCLC when modern medicine is utilized in conjunction with TCM treatment, thereby offering a foundation for the prognostic assessment of stage IIIB/IV NSCLC and guiding personalized treatment strategies for patients with NSCLC by utilizing a combination of TCM and Western medicine.

The present study showed that TCM treatment significantly affected the prognosis of patients diagnosed with advanced NSCLC. Additionally, these survival prediction models have demonstrated that incorporating TCM treatment time into nomograms yields superior predictive outcomes compared with those that exclude this variable. This result is in line with that of the previous research conducted by Wang et al. In their cohort study, they noted that the combination of TCM and EGFR-tyrosine kinase inhibitors (EGFR-TKIs) significantly extended PFS and OS among patients with NSCLC with EGFR mutations [20]. Furthermore, previous research discovered, through a prospective observational, non-interventional study that the prognostic impact of pure TCM surpassed that of platinum-based chemotherapy alone in patients diagnosed with stage IV lung adenocarcinoma [21].

The aforementioned findings collectively illustrate the potential significance of TCM treatment. TCM, characterized by its holistic approach, may exert multifaceted effects on cancer management, including the modulation of the immune system, inhibition of tumor growth, and an enhancement of the body's resilience against cancer-related fatigue and other side effects of conventional cancer therapies [22]. The synergistic interaction between TCM and targeted therapies, such as EGFR-TKIs, could potentially amplify therapeutic efficacy, thereby extending PFS and OS [23,24]. Furthermore, the phytochemical constituents of various TCM formulations may exert anticancer effects through multiple pathways, such as apoptosis induction, angiogenesis inhibition, and metastasis suppression [25,26].

In the present study, we found that WBCs, an inflammatory factor, play a crucial role in the development, growth, infiltration, and spread of tumors. As a result, WBC counts in patients with advanced NSCLC have been shown to have predictive value for prognosis when undergoing immunotherapy [27,28]. Moreover, most studies have consistently demonstrated that baseline WBC count exhibits a greater predictive capacity [29,30]. Liu et al. developed a survival prognostic model of NSCLC that consistently identified the *C*-reactive protein (albumin) index as a significant prognostic risk factor [31]. Significantly, while the majority of prior research has established a correlation between inflammatory cells and survival prognosis in NSCLC patients undergoing immunotherapy, our study broadens this understanding by highlighting the equally significant influence of inflammatory cells in other therapies. In addition to the aforementioned prognostic factors, the present study identified sex, EGFR mutations, adrenal metastases, and maintenance therapy as significant and influential predictors, a finding substantiated by previously published studies [[32], [33], [34], [35]].

Despite being the first prognostic model for NSCLC survival that incorporates TCM treatment and demonstrates favorable predictability and reliability, the present study has certain limitations. First, it was conducted retrospectively, thereby exhibiting inherent flaws attributable to selection bias. For example, patients who received pure TCM treatment had a median OS of merely 3.8 months; however, it is worth noting that this particular cohort only sought TCM treatment after experiencing unsuccessful outcomes with other treatment modalities. Furthermore, despite the use of data from three branch hospitals, the present was not strictly a multicenter study. This limitation restricts the diversity of our patient population, and in turn, the generalizability of our findings. Another critical factor is the influence of TCM practices, which vary significantly across healthcare settings and geographical regions. Variations in TCM treatment availability, practitioner expertise, and treatment standardization may affect the feasibility of implementing a nomogram in diverse clinical contexts. The potential unmeasured confounding factors should not be overlooked. The inherent challenges in standardizing TCM treatments, owing to their personalized nature and wide variability in formulations, and practices pose significant obstacles in replicating this model across different clinical environments. This variability underscores the need for rigorous methodological approaches to systematically evaluate the efficacy of TCM treatments systematically, and their integration into standardized cancer care protocols.

## 5 Conclusions

The nomograms developed in the present study demonstrated an objective and accurate ability to predict the 1-, 2-, and 3-year OS rates of patients with advanced NSCLC. Furthermore, the nomograms exhibited satisfactory performance in both internal and external validation processes. TCM has the potential to assist clinicians in prognosticating patient outcomes, tailoring personalized treatment plans, and capitalizing on its therapeutic advantages. The present study, however, did have certain limitations, necessitating future multicenter studies with larger sample sizes to enhance the accuracy of prognostic predictions.

## Ethics approval

This study was approved by the Ethics Committee of Guangdong Provincial Hospital of Chinese Medicine (approval number BE2018-133-01). The ethics committees granted an exemption from the requirement for informed consent owing to the retrospective nature of this study.

## Consent for publication

Not applicable.

## Funding

This work was supported by The Project of 10.13039/100012829Guangdong Provincial Hospital of Chinese Medicine (YN2016QL04, YN2022QN24), and Guangzhou University of Chinese Medicine (A1-2601-24-414-111 Z60).

## CRediT authorship contribution statement

**Yihong Liu:** Writing – review & editing, Supervision, Methodology, Funding acquisition, Conceptualization. **Haochuan Ma:** Writing – original draft, Formal analysis, Data curation. **Rui Zhou:** Software, Resources, Project administration, Formal analysis, Data curation. **Yadong Chen:** Visualization, Validation, Software. **Yanjuan Zhu:** Visualization, Validation, Data curation. **Xuesong Chang:** Visualization, Validation. **Jicai Chen:** Visualization, Supervision, Resources, Investigation. **Haibo Zhang:** Writing – review & editing, Supervision, Methodology, Conceptualization.

## Declaration of competing interest

The authors declare that they have no known competing financial interests or personal relationships that could have appeared to influence the work reported in this paper.
