# Supplementary material for: Nomogram-based prognostic tool for stage IIIB/IV non-small cell lung cancer patients undergoing traditional Chinese medicine treatment
Source: Heliyon. 2024 May 18;10(10):e31449. doi: 10.1016/j.heliyon.2024.e31449 (PMC11137507; doi:10.1016/j.heliyon.2024.e31449)
Supplement: Multimedia component 1 [file mmc1.docx]

**Figure S1** Analysis of cut-off points for TCM treatment time by X-tile


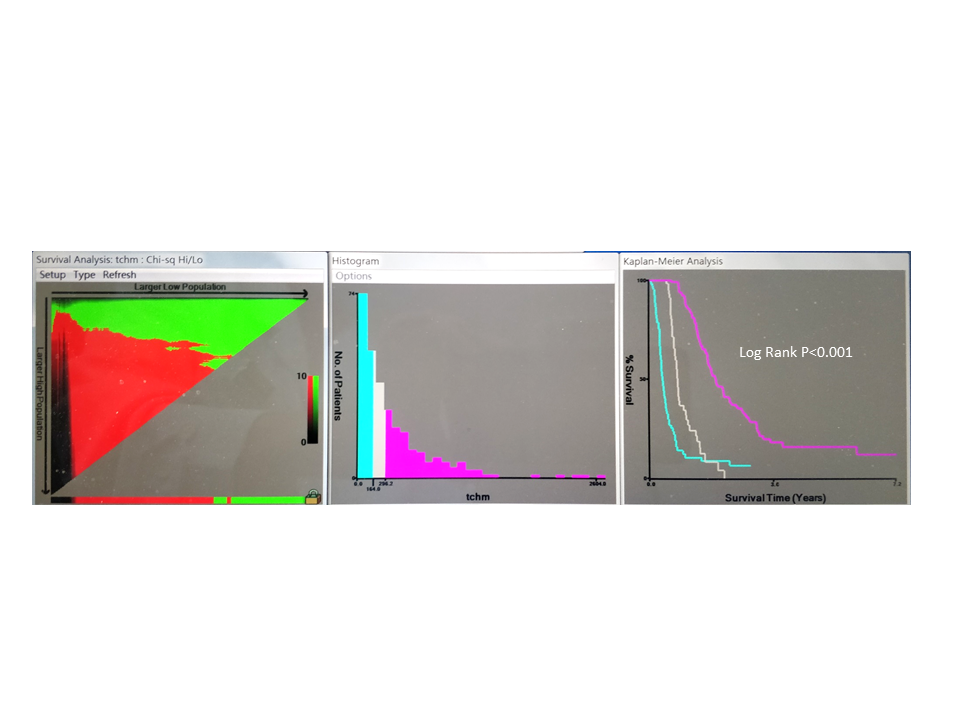


**Figure S2** Calibration curves for internal validation of nomogram A and B


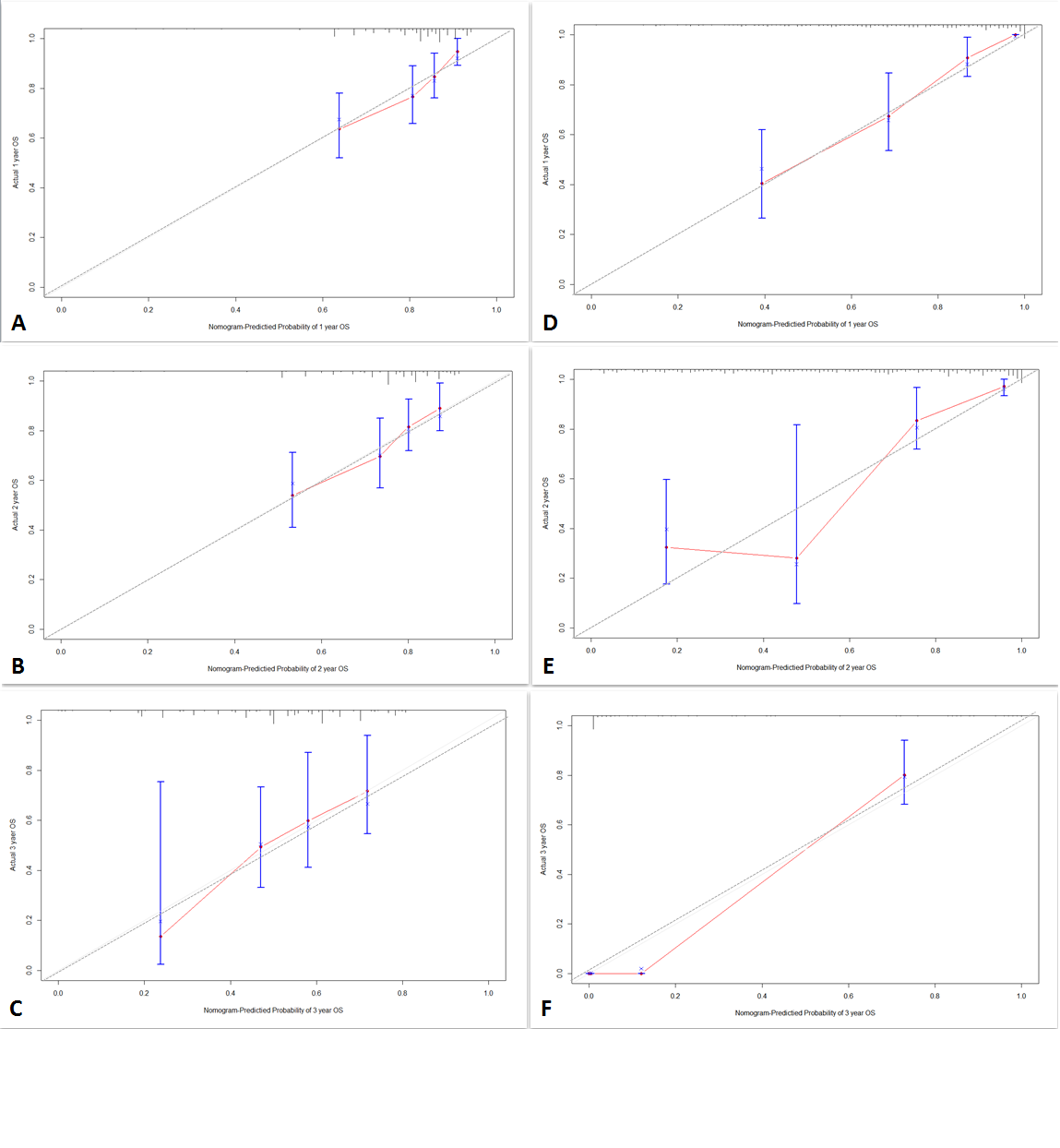


**Figure S3** Calibration curves for external validation of nomogram A and B


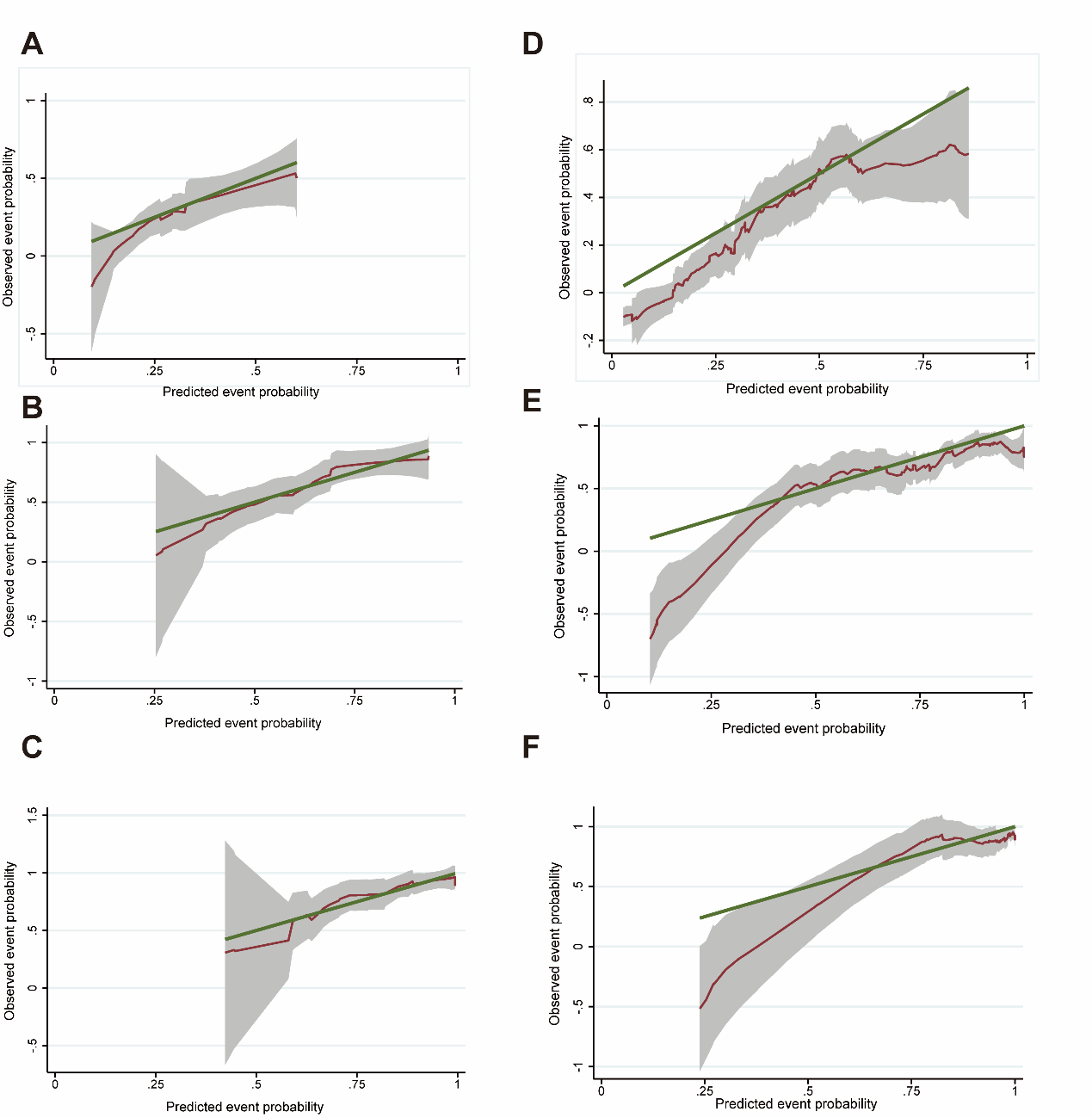


**Table S1** Detailed definitions of treatment-related variables

| **Predictor variables** | **Definitions** |
| --- | --- |
| Treatment regimen | A treatment regimen is defined as Western medical treatments, including chemotherapy, radiotherapy, targeted therapy, immunotherapy, and surgery. |
| Maintenance therapy | Maintenance therapy consists of prolonging the systemic antineoplastic treatment until progression or unacceptable toxicity. |
| Treatment line | Treatment line is the term used to describe the order in which different therapies are given to people as the tumor progresses. For example, first-line therapy is the first treatment given for cancer. |
| The regimen of TCM treatment | The regimen of TCM treatment refer to adjunctive TCM treatments used in Western medicine, including Chinese herbs, prescriptions, and acupuncture. |
| TCM treatment time | TCM treatment time is defined as the period from the beginning of receiving any TCM treatment to the end of the TCM treatment. |
